# Lasmiditan for acute treatment of migraine in patients with cardiovascular risk factors: post-hoc analysis of pooled results from 2 randomized, double-blind, placebo-controlled, phase 3 trials

**DOI:** 10.1186/s10194-019-1044-6

**Published:** 2019-08-29

**Authors:** Robert E. Shapiro, Helen M. Hochstetler, Ellen B. Dennehy, Rashna Khanna, Erin Gautier Doty, Paul H. Berg, Amaal J. Starling

**Affiliations:** 1 The University of Vermont Medical Center, 111 Colchester Ave, Burlington, VT 05401 USA; 2 Eli Lilly and Company, and/or one of its subsidiaries, Lilly Corporate Center, Indianapolis, IN 46285 USA; 3 Department of Psychological Sciences, Purdue University, 610 Purdue Mall, West Lafayette, IN 47907 USA; 4 Mayo Clinic, 13400 E. Shea Blvd, Scottsdale, AZ 85259 USA

**Keywords:** Migraine, Cardiovascular disease, Lasmiditan, Safety, Ditan

## Abstract

**Background:**

In addition to the increased risk for cardiovascular (CV) disease and CV events associated with migraine, patients with migraine can also present with a number of CV risk factors (CVRFs). Existing treatment options can be limited due to contraindications, increased burden associated with monitoring, or patient avoidance of side effects. Safe and effective migraine treatment options are needed for patients with migraine and a history of CV or cerebrovascular disease or with increased risk for CV events. This analysis was designed to evaluate the safety and efficacy of oral lasmiditan, a selective serotonin 5-hydroxytryptamine 1F receptor agonist, in acute treatment of migraine attacks in patients with CVRFs.

**Methods:**

SAMURAI and SPARTAN were similarly designed, Phase 3, randomized, double-blind, placebo-controlled trials in adults treating a single migraine attack with lasmiditan 50, 100, or 200 mg. Both studies included patients with CVRFs, and SPARTAN allowed patients with coronary artery disease, clinically significant arrhythmia, or uncontrolled hypertension. Efficacy and safety of lasmiditan in subgroups of patients with differing levels of CVRFs are reported. For efficacy analyses, logistic regression was used to assess treatment-by-subgroup interactions. For safety analyses, Cochran-Mantel-Haenszel test of general association evaluated treatment comparisons; Mantel-Haenszel odds ratio assessed significant treatment effects.

**Results:**

In this pooled analysis, a total of 4439 patients received ≥1 dose of study drug. A total of 3500 patients (78.8%) had ≥1 CVRF, and 1833 patients (41.3%) had ≥2 CVRFs at baseline. Both trials met the primary endpoints of headache pain freedom and most bothersome symptom freedom at 2 h. The presence of CVRFs did not affect efficacy results. There was a low frequency of likely CV treatment-emergent adverse events (TEAEs) overall (lasmiditan, 30 [0.9%]; placebo, 5 [0.4%]). There was no statistical difference in the frequency of likely CV TEAEs in either the absence or presence of any CVRFs. The only likely CV TEAE seen across patients with ≥1, ≥ 2, ≥ 3, or ≥ 4 CVRFs was palpitations.

**Conclusions:**

When analyzed by the presence of CVRFs, there was no statistical difference in lasmiditan efficacy or the frequency of likely CV TEAEs. Despite the analysis being limited by a single-migraine-attack design, the lack of differences in efficacy and safety with increasing numbers of CVRFs indicates that lasmiditan might be considered in the treatment algorithm for patients with CVRFs. Future studies are needed to assess long-term efficacy and safety.

**Trial registration:**

ClinicalTrials.gov NCT02439320 (SAMURAI), registered 18 March 2015 and ClinicalTrials.gov NCT02605174 (SPARTAN), registered 11 November 2015.

**Electronic supplementary material:**

The online version of this article (10.1186/s10194-019-1044-6) contains supplementary material, which is available to authorized users.

## Background

Migraine is a neurologic disease characterized by severe, intermittent headache attacks with associated symptoms including nausea, vomiting, phonophobia, and photophobia that can be chronic and disabling [1]. The disease can interfere significantly with occupational, educational, household, family, and social responsibilities [2]. It is the second largest cause of years lost to disability [3].

Migraine is an independent risk factor for cardiovascular (CV) disease [4, 5] and is associated with a number of CV events, including ischemic stroke, transient ischemic attack, ischemic heart disease, and myocardial infarction, as well as increased morbidity and mortality [6–11]. Although both migraine with and without aura are known to be associated with CV disease, these associations are more significant in patients with migraine with aura [5, 11–13]. A recent meta-analysis demonstrated that the presence of aura significantly affects the risk of stroke (adjusted hazard ratio [aHR] aura 1.56; 95% confidence interval [CI] 1.30–1.87 vs. aHR no aura 1.11; 95% CI 0.94–1.31; *p*_interaction_ = 0.01) [5]. In addition, statistical heterogeneity was lower for all CV and cerebrovascular outcomes when results were stratified by the presence of aura [5]. Existing options for acute migraine treatment may be contraindicated in patients with CV history or risk [14, 15]. For example, nonsteroidal anti-inflammatory drugs are associated with an increased risk of CV, thrombotic, and upper gastrointestinal events [16–18]. Due to vasoconstriction associated with the 5-hydroxytryptamine receptor 1B (5-HT_1B_) activity, triptans are contraindicated in patients with ischemic coronary artery disease (CAD), coronary artery vasospasm, Wolff-Parkinson-White syndrome, peripheral vascular disease, ischemic bowel disease, and uncontrolled hypertension and in patients with a history of cerebrovascular ischemic events [19–25]. Approximately 2 million women and 665,000 men in the United States have episodic migraine and a history of ≥1 CV event, condition, or procedure that may limit the use of triptans [26].

The desire to discover effective migraine treatments without vasoconstrictive properties led to the development of selective 5-hydroxytryptamine receptor 1F (5-HT_1F_) agonists and other molecules [27–33]. Lasmiditan selectively targets 5-HT_1F_ receptors on neurons in the central and peripheral trigeminal system, decreasing neuropeptide release and inhibiting pain pathways, including the trigeminal nerve [34, 35]. Preclinical studies have demonstrated that messenger RNA for 5-HT_1F_ receptors is highly expressed in human middle cerebral arteries [36] and human coronary arteries [37], but in vitro studies suggest that 5-HT_1F_ receptors do not mediate significant vasoconstriction effects in human cerebral or coronary vessels [37–40]. Data from nonclinical animal and in vitro studies indicate that lasmiditan does not cause vasoconstriction in coronary, carotid, and internal mammary arteries [35, 41, 42].

The efficacy and safety of oral lasmiditan in the acute treatment of migraine attacks have been demonstrated in 2 randomized, double-blind, placebo-controlled, Phase 3 studies, SAMURAI and SPARTAN [43, 44]. This publication reports the pooled safety and efficacy of lasmiditan in a subpopulation of patients with CV risk factors (CVRFs).

## Methods

### Patients and study design

Detailed design and clinical results of SAMURAI and SPARTAN have been reported [43, 44]. SAMURAI and SPARTAN shared many study design elements, allowing for integrated analyses. Briefly, both trials were randomized, double-blind, placebo-controlled, Phase 3 studies of a single migraine attack. These studies were conducted in accordance with the principles of the Declaration of Helsinki. The institutional review board or independent ethics committee at each site approved the protocols, and all patients provided written informed consent. All authors had access to the study data and have reviewed and approved the final manuscript. SAMURAI and SPARTAN were conducted in patients with migraine with and without aura (based on history alone), with the primary objective of evaluating the efficacy of lasmiditan versus placebo as measured by the proportion of patients who became headache pain-free and most bothersome symptom (MBS)-free at 2 h. Patients identified their MBS from nausea, photophobia, or phonophobia at baseline. Patients were randomized to a double-blind, 2-dose sequence of oral lasmiditan 200 mg, 100 mg, or 50 mg (SPARTAN only) or placebo (in equal proportions for the first dose); patients were allowed to take a second dose of study drug of the same strength 2 to 24 h after the first dose if symptoms persisted or returned. For the second dose, the placebo arm received placebo and active treatment arms received either the same strength of lasmiditan or placebo (2:1 ratio).

#### Inclusion/exclusion criteria

The 2 trials enrolled very similar populations. However, SPARTAN allowed enrollment of patients with known CAD, clinically significant arrhythmia, or uncontrolled hypertension, whereas such patients were excluded in SAMURAI.

#### Baseline cardiovascular/cerebrovascular-related history

A patient was identified as having baseline CV/cerebrovascular-related history (CCRH) if the patient self-reported 1 or more conditions included in the narrow search terms of the following Standardized Medical Dictionary for Drug Regulatory Activities (MedDRA) Queries (SMQs): Cardiac arrhythmias, Cardiac failure, Cardiomyopathy, Central nervous system (CNS) vascular disorders, Embolic and thrombotic events, Hypertension, Ischemic heart disease, Pulmonary hypertension, and Torsade de pointes/QT prolongation.

#### Concomitant cardiovascular medications

CV medications were identified using the World Health Organization’s Anatomical Therapeutic Chemical/Defined Daily Dose codes within the “Cardiac System” and “Antithrombotic Agents.” The selected medications were then reviewed to confirm that the indication for use was a CV condition (per medical history or adverse event [AE]). For example, CV medications being used for migraine prevention (eg, beta blockers and calcium channel blockers) as the indication were removed.

### Identification of cardiovascular risk factors

For the pooled analyses of the primary objectives and safety measures, CVRFs of interest included the 6 variables that the American College of Cardiology/American Heart Association Task Force on Practice Guidelines concluded were the most robust variables for prediction of a first CV event [45]. A present/absent criterion was applied to each variable to assess the proportion of patients with each potential risk. The variables and their defined thresholds were as follows: age > 40 years [45], self-report of diabetes diagnosis, current smoker, baseline total cholesterol ≥240 mg/dL [45], baseline high-density lipoprotein cholesterol < 40 mg/dL for men or <  50 mg/dL for women [46], and baseline systolic blood pressure ≥ 140 mmHg [47] and/or self-reported medical history of high blood pressure at baseline.

Subgroup efficacy analyses compared patients with ≥2 CVRFs to those with 0 or 1 CVRF(s), since many patients accrued 1 risk factor based on the age variable alone. Analyses of safety measures were performed based on the number of CVRFs, with categories of 0, ≥ 1, ≥ 2, ≥ 3, and ≥ 4 risk factors.

### Study evaluations and analyses

For efficacy evaluations, the proportions of patients achieving headache pain freedom and MBS freedom at 2 h after the first dose were compared in lasmiditan- and placebo-treated groups.

For safety evaluations, treatment-emergent adverse events (TEAEs), defined as events that initially occurred or worsened in severity after the first dose of study drug and occurred within 48 h of dose, were analyzed. AEs irrespective of temporal association with dosing were also analyzed because some CV events may have been identified at a later time (eg, during laboratory, vital signs, and/or electrocardiogram [ECG] assessments). Dose groups in the tables show the dose that the patients were randomized to; if the patient took a second dose of lasmiditan, their total dose may have been higher.

Potential CV AEs were identified by querying the full list of AEs for specific terms within the following SMQs: Cardiac arrhythmias, Cardiac failure, Cardiomyopathy, CNS vascular disorders, Embolic and thrombotic events, Hypertension, Ischemic heart disease, Pulmonary hypertension, and Torsade de pointes/QT prolongation along with the Preferred Terms (PTs) abdominal pain, abdominal pain upper, and abdominal pain lower. SMQs are validated, predetermined sets of MedDRA terms grouped together to aid with safety analyses and reporting. SMQs are independent of each other, and some terms could overlap between SMQs.

The resultant listing of potential CV AEs was then reviewed by a group of unblinded Eli Lilly physicians to determine which were likely CV in nature. For example, if an AE of “edema” occurred in close association with a local injury, then it was not considered a likely CV AE. The events determined to represent likely CV events (AEs and TEAEs) are discussed in detail.

### Statistical analysis

Data handling rules and full analysis methods were previously described in Kuca et al. [43] and Wietecha et al. [44]. Efficacy analyses were conducted in the modified Intent-to-Treat population consisting of patients who took study drug within 4 h of migraine attack onset and had at least 1 postdose efficacy assessment. Safety and tolerability analyses were conducted in the Safety population consisting of patients who took study drug. For the analyses of headache pain freedom and MBS freedom in subgroups by number of CVRFs, the *p* value was calculated for treatment-by-subgroup interaction, based on logistic regression with terms for study, subgroup, treatment, and treatment-by-subgroup in the model.

AEs were classified based on MedDRA version 21.0. Missing dates and times for dosing and AEs were imputed to avoid underestimation of frequency or duration of AEs and to increase the sensitivity of identifying TEAEs. The number and percentage of patients who reported TEAEs were summarized, and the results are presented by decreasing frequency of PTs in the all lasmiditan dose group.

Statistical comparisons were made between all lasmiditan doses combined and placebo as follows, unless otherwise noted. First, the Cochran-Mantel-Haenszel test of general association stratified by study was used for treatment comparisons of percentages. In addition to the Cochran-Mantel-Haenszel test, the Mantel-Haenszel odds ratio (OR) and the flag of *p* value < 0.1 Breslow-Day test for homogeneity of OR are displayed. ORs were created with treatment as the numerator and placebo as the denominator. In addition, study size-adjusted percentages are provided using the methodology of Crowe et al. [48].

Tests with 2-sided *p* values less than 0.05 are referred to as having statistical significance for a treatment difference, unless otherwise noted. However, *p* values should not be overinterpreted for safety analyses. Except for prespecified hypotheses, they correspond to data-driven hypotheses and, hence, are only useful as a flagging mechanism.

## Results

### Baseline patient characteristics

Across both trials, a total of 4439 patients took ≥1 dose of study drug, and 3701 patients were in the modified Intent-to-Treat population. The proportion of each CVRF is reported in Table 1. The distribution of the CVRFs (across all 6 factors) was balanced between the lasmiditan- and placebo-treated patients and across the lasmiditan dose groups (data not shown). The majority of patients (78.8%) had ≥1 CVRF (Table 1) with similar results between lasmiditan- and placebo-treated patients (Table 2). Overall, the frequencies of patients with 0, 1, 2, 3, 4, or 5 CVRFs were balanced across the lasmiditan- and placebo-treated groups (Table 2). There were no patients with 6 CVRFs. Baseline CCRH was reported in 20.4% of patients. The most frequently reported types of CCRH were hypertension (*n* = 719), angina pectoris (*n* = 24), deep vein thrombosis (*n* = 16), myocardial infarction (*n* = 14), pulmonary embolism (*n* = 14), transient ischemic attack (*n* = 13), and CAD (*n* = 13). A small number of patients had contraindications to the use of a triptan (*n* = 15 in SAMURAI; *n* = 75 in SPARTAN).

**Table 1:** Summary of cardiovascular risk factors, other risk factors, and laboratory values and vital signs at baseline by sex

Characteristic (unit)	Females (*N* = 3726) *n* (%)	Males (*N* = 713) *n* (%)	Pooled (*N* = 4439) *n* (%)
CVRFs per ACC/AHA recommended variables^a^			
Age > 40 years	2044 (54.9)	387 (54.3)	2431 (54.8)
Current smoker	490 (13.2)	139 (19.5)	629 (14.2)
High total cholesterol (≥ 240 mg/dL)	421 (11.3)	70 (9.8)	491 (11.1)
Low HDL cholesterol (< 40 mg/dL in men, < 50 mg/dL in women)	1197 (32.1)	206 (28.9)	1403 (31.6)
High blood pressure (SBP ≥ 140 mmHg and/or medical history of hypertension at baseline)	775 (20.8)	200 (28.1)	975 (22.0)
Medical history of diabetes mellitus, total	215 (5.8)	53 (7.4)	268 (6.0)
Type 1	8 (0.2)	2 (0.3)	10 (0.2)
Type 2	154 (4.1)	31 (4.3)	185 (4.2)
Type unspecified	53 (1.4)	20 (2.8)	73 (1.6)
Number of CVRFs			
≥1	2939 (78.9)	561 (78.7)	3500 (78.8)
≥2	1507 (40.4)	326 (45.7)	1833 (41.3)
≥3	545 (14.6)	125 (17.5)	670 (15.1)
≥4	133 (3.6)	36 (5.0)	169 (3.8)
≥5	18 (0.5)	7 (1.0)	25 (0.6)
≥6	0 (0.0)	0 (0.0)	0 (0.0)
Other risk factors of potential interest			
Postmenopausal	573 (15.4)	N/A	N/A
Obese (BMI ≥ 30 kg/m^2^)	1655 (44.4)	278 (39.0)	1933 (43.5)
History of migraine with aura	1495 (40.1)	264 (37.0)	1759 (39.6)
High LDL cholesterol (≥ 160 mg/dL)	245 (6.6)	46 (6.5)	291 (6.6)
Medical history of hypertension	569 (15.3)	134 (18.8)	703 (15.8)
Family history of CAD	1134 (30.4)	160 (22.4)	1294 (29.2)
Laboratory values and vital signs, mean (SD)			
Total cholesterol (mg/dL)	192.0 (39.4)	190.6 (38.3)	191.8 (39.3)
HDL cholesterol	58.0 (16.1)	48.3 (14.6)	56.4 (16.2)
LDL cholesterol	107.7 (33.3)	109.9 (32.8)	108.1 (33.3)
SBP (mm Hg)	120.0 (13.9)	127.5 (12.9)	121.2 (14.0)

^a^ACC/AHA guideline-recommended variables for CV risk assessment in adults without diagnosed disease [45]

*ACC/AHA* American College of Cardiology/American Heart Association, *BMI* Body mass index, *CAD* Coronary artery disease, *CV* Cardiovascular, *CVRF* Cardiovascular risk factor, *HDL* High-density lipoprotein, *LDL* Low-density lipoprotein, *N* total number of patients in each group, *n* number of patients with risk factor, *N/A* Not applicable, *SBP* Systolic blood pressure, *SD* Standard deviation

**Table 2:** Frequency of cardiovascular risk factors by dose regimen

Number of CVRFs^a^	Placebo *N* = 1262 *n* (%)	All LTN *N* = 3177 *n* (%)	Total *N* = 4439 *n* (%)
0	255 (20.2)	684 (21.5)	939 (21.2)
1	486 (38.5)	1181 (37.2)	1667 (37.6)
2	326 (25.8)	837 (26.3)	1163 (26.2)
3	144 (11.4)	357 (11.2)	501 (11.3)
4	43 (3.4)	101 (3.2)	144 (3.2)
5	8 (0.6)	17 (0.5)	25 (0.6)

^a^CVRFs were based on the American College of Cardiology/American Heart Association Task Force on Practice Guidelines [45] and included age, total and high-density lipoprotein cholesterol, systolic blood pressure (including treated or untreated status), diabetes, and current smoking status

*CVRFs* Cardiovascular risk factors, *LTN* Lasmiditan, *N* number of patients in the analysis population, *n* number of patients within each specific category

A total of 21.8% of patients reported concomitant use of CV medication for reasons other than migraine at baseline. There was no statistical difference between treatment groups in the proportions of patients taking concomitant CV medicines either overall or for any medication class. The most commonly used medications in either of the treatment groups were agents acting on the renin-angiotensin system (*n* = 393), lipid-modifying agents (*n* = 386), and beta-blocking agents (*n* = 231). Examples of other medications used were diuretics, antithrombotics, calcium channel blockers, and other cardiac therapies such as cardiac glycosides and antiarrhythmics.

### Efficacy

**Fig. 1:**
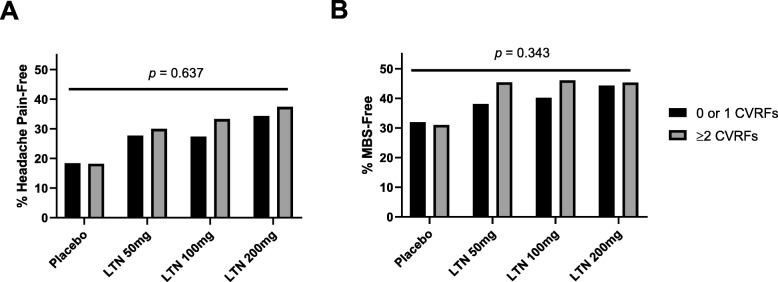
Proportion of patients in the mITT population who were headache pain-free (**a**) and MBS-free (**b**) at 2 h by the degree of cardiovascular risk. *CVRF* cardiovascular risk factor, *LTN* lasmiditan, *MBS* most bothersome symptom, *mITT* modified Intent-to-Treat. Note: *p* values are for treatment-by-subgroup interaction, based on logistic regression with terms for study, subgroup, treatment, and treatment by-subgroup in the model

Both studies met the primary objective, with significantly more lasmiditan-treated patients headache pain-free as well as MBS-free at 2 h at all doses compared with placebo-treated patients [43, 44]. Within pooled subgroups of patients with 0 or 1 CVRF(s) and with ≥2 CVRFs, the proportion of patients who were pain-free and MBS-free at 2 h were unaffected by the degree of CV risk (Fig. 1).

### Safety and tolerability

#### Medical review of potential adverse events

Prior to medical review, 3.1% (*n* = 97) of lasmiditan-treated patients and 1.4% (*n* = 18) of placebo-treated patients were noted to have at least 1 potential CV AE, reported irrespective of time following dosage. Significant, although rare, reports of events were coded by SMQ as cardiomyopathy (0.8% [*n* = 25] of lasmiditan vs. 0.2% [*n* = 2] of placebo [OR = 5.08, *p* = 0.01]) and CNS vascular disorders (0.3% [*n* = 11] of lasmiditan vs. 0.0% [*n* = 0] of placebo [*p* = 0.03]). Following medical review, 1.7% (*n* = 55) of lasmiditan-treated and 1.3% (*n* = 16) of placebo-treated patients were considered to have at least 1 likely CV AE. A total of 44 cases were excluded (Additional file 1: Table S1), about half of which were due to abdominal pain with no other CV-related symptoms. Other examples of exclusion were due to events of shortness of breath or syncope (coded by SMQ as “cardiomyopathy”) or dysarthria (coded by SMQ as “CNS vascular disorders”) without having a history of these events, a comorbid cardiac event, or a concomitant CV medication. Details of the potential and likely CV AEs are presented in Additional file 1: Table S2 and Table S3, respectively, and are discussed in more detail in the Additional files.

#### Likely CV treatment-emergent adverse events

The number and percentage of patients with at least 1 likely CV TEAE, although not statistically significant, were higher in those treated with lasmiditan (*n* = 30 [0.9%]) than in those treated with placebo (*n* = 5 [0.4%]) (Table 3). In the Cardiac arrhythmias SMQ, a significantly greater number of events were reported in the lasmiditan-treated group, largely due to reports of palpitations, tachycardia, and increased heart rate. All events were mild to moderate.

**Table 3:** Summary and analysis of likely cardiovascular treatment-emergent adverse events

Standardized MedDRA Query Preferred Term	Placebo (*N* = 1262)	All LTN (*N* = 3177)	Comparison between all LTN vs. placebo		
	*n* (%) [adj %]	*n* (%) [adj %]	OR^a^	95% CI^a^	*p* value^b^
Patients with at least 1 likely CV TEAE	5 (0.4) [0.4]	30 (0.9) [0.9]	2.46	(0.95, 6.39)	0.06
Cardiac arrhythmias (SMQ)	3 (0.2) [0.2]	27 (0.8) [0.9]	3.59	(1.09, 11.79)	**0.02**
Palpitations	1 (0.1) [0.1]	12 (0.4) [0.4]	4.67	(0.63, 34.69)	0.09
Tachycardia	0 (0.0) [0.0]	6 (0.2) [0.2]			0.14
Heart rate increased	1 (0.1) [0.1]	5 (0.2) [0.2]	1.89	(0.23, 15.65)	0.54
Bradycardia	1 (0.1) [0.1]	1 (0.0) [0.0]	0.50		0.62
Electrocardiogram abnormal	0 (0.0) [0.0]	1 (0.0) [0.0]			0.48
Sinus bradycardia	0 (0.0) [0.0]	1 (0.0) [0.0]			0.48
Syncope	0 (0.0) [0.0]	1 (0.0) [0.0]			0.56
Cardiomyopathy (SMQ)	1 (0.1) [0.1]	14 (0.4) [0.4]	5.45	(0.74, 40.05)	0.06
Palpitations	1 (0.1) [0.1]	12 (0.4) [0.4]	4.67	(0.63, 34.69)	0.09
Electrocardiogram abnormal	0 (0.0) [0.0]	1 (0.0) [0.0]			0.48
Syncope	0 (0.0) [0.0]	1 (0.0) [0.0]			0.56
Hypertension (SMQ)	0 (0.0) [0.0]	3 (0.1) [0.1]			0.28
Hypertension	0 (0.0) [0.0]	2 (0.1) [0.1]			0.36
Blood pressure increased	0 (0.0) [0.0]	1 (0.0) [0.0]			0.56
Pulmonary hypertension (SMQ)	1 (0.1) [0.1]	0 (0.0) [0.0]	0.00		0.16
Cardiac murmur	1 (0.1) [0.1]	0 (0.0) [0.0]	0.00		0.16
Torsade de pointes/QT prolongation (SMQ)	0 (0.0) [0.0]	1 (0.0) [0.0]			0.56
Syncope	0 (0.0) [0.0]	1 (0.0) [0.0]			0.56
Any abdominal pain (PT)	1 (0.1) [0.1]	0 (0.0) [0.0]	0.00		0.16
Abdominal pain upper	1 (0.1) [0.1]	0 (0.0) [0.0]	0.00		0.16

^a^Mantel-Haenszel OR stratified by study and 95% CI (CI calculated if ≥4 events in numerator and ≥ 1 event in denominator)

^b^*p* values are from Cochran-Mantel-Haenszel test of general association stratified by study. Bold indicates a *p* value < 0.05

*adj %* study size adjusted percentage, *CI* Confidence interval, *CNS* Central nervous system, *CV* Cardiovascular, *LTN* Lasmiditan, *MedDRA* Medical Dictionary for Drug Regulatory Activities, *N* Number of patients in the analysis population, *n* number of patients within each specific category, *OR* Odds ratio, *PT* Preferred Term, *SMQ* Standardized MedDRA Query, *TEAE* treatment-emergent adverse event

Likely CV TEAEs are from medical review out of potential CV TEAEs that are selected based on broad and narrow terms in the SMQs Cardiac arrhythmias, Cardiac failure, Cardiomyopathy, CNS vascular disorders, Embolic and thrombotic events, Hypertension, Ischemic heart disease, Pulmonary hypertension, and Torsade de pointes/QT prolongation and the PTs abdominal pain, abdominal pain upper, and abdominal pain lower

Any abdominal pain (PT) consists of the PTs abdominal pain, abdominal pain upper, and abdominal pain lower

MedDRA version 21.0

There were no discontinuations due to likely CV TEAEs. No deaths were reported in any patients who took study drug or placebo. One lasmiditan-treated patient had a serious TEAE of worsening hypertension. The patient had preexisting hypertension, which was under treatment at screening with nifedipine 100 mg. The patient was hospitalized and symptoms resolved following an increase in nifedipine dose to 150 mg. The patient had normal blood pressure during screening (120/84 mmHg) and at the end of study visit (110/74 mmHg).

#### Likely CV treatment-emergent adverse events by CVRF categories

Table 4 shows likely CV TEAEs when analyzed by the number of CVRFs. There was no statistical difference in the frequency of likely CV TEAEs either in the absence or presence of any CVRFs between the placebo and pooled lasmiditan treatment groups at either an SMQ or an individual PT level. The only likely CV TEAE seen across patients with ≥1, ≥ 2, ≥ 3, or ≥ 4 CVRFs was palpitations. No statistical dose response between the lasmiditan 100 and 200 mg doses was observed for any of the likely CV TEAEs based on the CVRFs overall, with increasing number of risk factors, or for individual TEAEs. There were numerically more patients with a likely CV TEAE in the pooled lasmiditan 200-mg (*n* = 14 [1.1%]) and 100-mg dose groups (*n* = 13 [1.0%]) compared with the 50-mg dose group (*n* = 3 [0.5%]). The 50-mg dose group was not included in the pooled test of trend because the 50-mg dose was included only in one of the studies.

**Table 4:** Summary and analysis of likely cardiovascular treatment-emergent adverse events by cardiovascular risk factor categories

Categorical baseline CV risk factors Preferred Term	Placebo (*N* = 1262)	All LTN (*N* = 3177)	Comparison between all LTN vs. placebo		
	*n* (%) [adj %]	*n* (%) [adj %]	OR^a^	95% CI^a^	*p* value^b^
0	0 (0.0) [0.0]	5 (0.2) [0.2]			0.14
Bradycardia	0 (0.0) [0.0]	1 (0.0) [0.0]			0.48
Hypertension	0 (0.0) [0.0]	1 (0.0) [0.0]			0.56
Palpitations	0 (0.0) [0.0]	1 (0.0) [0.0]			0.48
Sinus bradycardia	0 (0.0) [0.0]	1 (0.0) [0.0]			0.48
Tachycardia	0 (0.0) [0.0]	1 (0.0) [0.0]			0.56
≥ 1	5 (0.4) [0.4]	25 (0.8) [0.8]	2.03	(0.77, 5.34)	0.15
Palpitations	1 (0.1) [0.1]	11 (0.3) [0.3]	4.22	(0.56, 31.70)	0.12
Heart rate increased	1 (0.1) [0.1]	5 (0.2) [0.2]	1.89	(0.23, 15.65)	0.54
Tachycardia	0 (0.0) [0.0]	5 (0.2) [0.2]			0.18
Blood pressure increased	0 (0.0) [0.0]	1 (0.0) [0.0]			0.56
Electrocardiogram abnormal	0 (0.0) [0.0]	1 (0.0) [0.0]			0.48
Hypertension	0 (0.0) [0.0]	1 (0.0) [0.0]			0.48
Syncope	0 (0.0) [0.0]	1 (0.0) [0.0]			0.56
Abdominal pain upper	1 (0.1) [0.1]	0 (0.0) [0.0]	0.00		0.16
Bradycardia	1 (0.1) [0.1]	0 (0.0) [0.0]	0.00		0.16
Cardiac murmur	1 (0.1) [0.1]	0 (0.0) [0.0]	0.00		0.16
≥ 2	3 (0.2) [0.2]	11 (0.3) [0.3]	1.48	(0.41, 5.38)	0.56
Palpitations	0 (0.0) [0.0]	4 (0.1) [0.1]			0.20
Tachycardia	0 (0.0) [0.0]	3 (0.1) [0.1]			0.28
Heart rate increased	1 (0.1) [0.1]	2 (0.1) [0.1]	0.67		0.74
Blood pressure increased	0 (0.0) [0.0]	1 (0.0) [0.0]			0.56
Hypertension	0 (0.0) [0.0]	1 (0.0) [0.0]			0.48
Abdominal pain upper	1 (0.1) [0.1]	0 (0.0) [0.0]	0.00		0.16
Cardiac murmur	1 (0.1) [0.1]	0 (0.0) [0.0]	0.00		0.16
≥ 3	0 (0.0) [0.0]	3 (0.1) [0.1]			0.28
Hypertension	0 (0.0) [0.0]	1 (0.0) [0.0]			0.48
Palpitations	0 (0.0) [0.0]	1 (0.0) [0.0]			0.56
Tachycardia	0 (0.0) [0.0]	1 (0.0) [0.0]			0.56
≥ 4	0 (0.0) [0.0]	1 (0.0) [0.0]			0.56
Palpitations	0 (0.0) [0.0]	1 (0.0) [0.0]			0.56

^a^Mantel-Haenszel OR stratified by study and 95% CI (CI calculated if ≥4 events in numerator and ≥ 1 event in denominator)

^b^*p* values are from Cochran-Mantel-Haenszel test of general association stratified by study

*ACC/AHA* American College of Cardiology and American Heart Association, *adj %* study size adjusted percentage, *CI* confidence interval, *CNS* central nervous system, *CV* cardiovascular, *HDL* high-density lipoprotein, *LTN* lasmiditan, *MedDRA* Medical Dictionary for Drug Regulatory Activities, *N* number of patients in the analysis population, *n* number of patients within each specific category, *OR* odds ratio, *PT* Preferred Term, *SMQ* Standardized MedDRA Query, *TEAE* treatment-emergent adverse event

Note: Likely CV TEAEs are from medical review out of potential CV TEAEs that are selected based on broad and narrow terms in the SMQs Cardiac arrhythmias, Cardiac failure, Cardiomyopathy, CNS vascular disorders, Embolic and thrombotic events, Hypertension, Ischemic heart disease, Pulmonary hypertension, and Torsade de pointes/QT prolongation and the PTs abdominal pain, abdominal pain upper, and abdominal pain lower

The CV disease risk factors are identified based on the ACC/AHA Task Force on Practice Guidelines [45]. A present/absent criterion was applied to each variable as follows: age > 40 years for both men and women, diabetes mellitus (any), current smoker, total cholesterol ≥240 mg/dL (laboratory measure), HDL cholesterol < 40 mg/dL for men and < 50 mg/dL for women (laboratory measure), and systolic blood pressure ≥ 140 mmHg (vital signs measure) and/or self-reported high blood pressure were included as hypertension

MedDRA version 21.0

#### Likely CV adverse events and CV treatment-emergent adverse events by history of aura

Given that aura is a potential factor that may increase the risk of CV events, likely CV AEs and TEAEs were summarized for patients with and without a history of aura for placebo and lasmiditan treatment groups. Likely CV AEs and likely CV TEAEs were similar regardless of history of aura (Additional file 1: Table S4 and Table S5, respectively) for both placebo- and lasmiditan-treated groups.

## Discussion

Options for acute treatment of migraine attacks are limited in patients with prior history of CV and cerebrovascular diseases and CVRFs, which are a significant percentage of the migraine patient population especially with increasing age. Migraine itself is a risk factor for CV disease and CV events, and these associations are more significant in patients with aura. In addition, patients may have other risk factors including hypertension and diabetes. Finding a treatment that does not exacerbate these risks could improve safety over existing treatments such as triptans, which are contraindicated in patients with CV history or risk.

Lasmiditan is a centrally penetrant, non-vasoconstrictive, selective 5-HT_1F_ receptor agonist being developed for the acute treatment of migraine. Lasmiditan inhibits trigeminovascular nociception by activation of 5-HT_1F_ receptors [36]. The purpose of this analysis was to examine the safety and efficacy of lasmiditan in patients with CVRFs from two Phase 3 studies, SAMURAI and SPARTAN.

This pooled Phase 3 population included a well-balanced population across lasmiditan- and placebo-treated patients with respect to the presence of baseline CCRH (approximately 20%) and 1 or more CVRFs (79% with ≥1, 41% with ≥2, and 15% with ≥3 CVRFs) in addition to their migraine history. The rates of risk factors appear to be generally representative of the overall migraine population; for example, in the American Migraine Prevalence and Prevention study, patients with migraine with ≥1, ≥ 2, and ≥ 3 risk factors are numbered 70%, 40%, and 19%, respectively [26].

Headache pain freedom and MBS freedom at 2 h examined by subgroups of 0 or 1 compared to ≥2 CVRFs were not significantly different in any dose regimen of lasmiditan, indicating that lasmiditan efficacy is not affected by the presence of CVRFs. In general, a small number of CV AEs including TEAEs were reported in the placebo-controlled studies. There were no ischemic CV TEAEs reported. There was no statistical difference between placebo and lasmiditan in the frequency of likely CV TEAEs either in the absence or presence of CVRFs at either an SMQ or an individual PT level. The only likely CV TEAE seen across patients with ≥1, ≥ 2, ≥ 3, or ≥ 4 CVRFs was palpitations. The subjective AE descriptor palpitations (which includes tachycardia and increased heart rate) is considered an adverse drug reaction with lasmiditan; however, the reported incidence was < 1%. Since concomitant vital signs or ECGs were not recorded during these symptoms, it is not known whether they were associated with true increases or decreases in heart rate. In clinical pharmacology studies using objective measures, lasmiditan was associated with decreases in heart rate of − 5 to − 10 beats per minute following doses of 50 to 200 mg (data unpublished). Additionally, the symptom of palpitations could reflect CV changes or actually wholly non-cardiac sources, such as anxiety or panic. In most of the cases, there was a report of other concurrent TEAEs, mostly neurological. Although the mechanism of action is unknown for which lasmiditan may cause palpitations or a decrease in heart rate, caution is advised for concomitant use of lasmiditan with other drugs that may lower heart rate.

Limitations of these analyses include the small sample size in patients with CCRH, the single migraine attack design, the medical review of potential CV AEs was performed by unblinded Lilly physicians, and the lack of vital sign and ECG measurements around the time of dosing. Additionally, potential rare events require large sample sizes and longer duration of observation than was available in these single-attack studies. Despite the limitations, these studies provide insight into the efficacy and safety of lasmiditan in patients with CVRFs. Results of multiple-attack studies (such as NCT03670810 and NCT02565186) will provide insight into efficacy and safety over time.

## Conclusions

We found that the proportion of patients achieving headache pain freedom and MBS freedom at 2 h were similar within subgroups of patients with 0 or 1 CVRF(s) and with ≥2 CVRFs, indicating that lasmiditan efficacy is not affected by the presence of CVRFs. We also found no statistical difference between placebo and lasmiditan in the frequency of likely CV TEAEs either in the absence or presence of any CVRFs in these single-attack studies. The lack of differences in efficacy and safety with increasing numbers of CVRFs indicates that lasmiditan might be considered in the treatment algorithm for patients with CVRFs. Longer-term studies are needed to evaluate efficacy and safety over time.

## Additional file

Additional file 1: Supplemental materials. **Table S1.** Summary of events excluded from likely adverse events and the associated Standardized MedDRA Queries/Preferred Terms. **Table** ***S*2*.*** Summary and analysis of potential cardiovascular adverse events within Standardized MedDRA Queries/Preferred Terms. **Table S3.** Summary and analysis of likely cardiovascular adverse events within Standardized MedDRA Queries/Preferred Terms**. Table S4.** Summary of likely cardiovascular adverse events within Standardized MedDRA Queries/Preferred Terms in patients with and without aura. **Table S5.** Summary of likely cardiovascular treatment-emergent adverse events within Standardized MedDRA Queries/Preferred Terms in patients with and without aura. (DOCX 39 kb)

## Data Availability

The datasets used and/or analyzed during the current study are available from the corresponding author upon reasonable request.

## Abbreviations

5-HT_1B_: 5-hydroxytrptamine receptor 1B
5-HT_1F_: 5-hydroxytryptamine receptor 1F
AE: Adverse event
aHR: adjusted hazard ratio
CAD: Coronary artery disease
CCRH: Cardiovascular/cerebrovascular-related history
CI: Confidence interval
CNS: Central nervous system
CV: Cardiovascular
CVRF: Cardiovascular risk factor
ECG: Electrocardiogram
MBS: Most bothersome symptom
MedDRA: Medical Dictionary for Drug Regulatory Activities
OR: Odds ratio
PT: Preferred Term
SMQ: Standardized Medical Dictionary for Drug Regulatory Activities Query
TEAE: Treatment-emergent adverse event
