# Supplementary material for: Lasmiditan for acute treatment of migraine in patients with cardiovascular risk factors: post-hoc analysis of pooled results from 2 randomized, double-blind, placebo-controlled, phase 3 trials
Source: J Headache Pain. 2019 Aug 29;20(1):90. doi: 10.1186/s10194-019-1044-6 (PMC6734241; doi:10.1186/s10194-019-1044-6)
Supplement: Supplementary file 1 — Supplemental materials. Table S1. Summary of events excluded from likely adverse events and the associated Standardized MedDRA Queries/Preferred Terms. Table S2. Summary and analysis of potential cardiovascular adverse events within Standardized MedDRA Queries/Preferred Terms. Table S3. Summary and analysis of likely cardiovascular adverse events within Standardized MedDRA Queries/Preferred Terms. Table S4. Summary of likely cardiovascular adverse events within Standardized MedDRA Queries/Preferred Terms in patients with and without aura. Table S5. Summary of likely cardiovascular treatment-emergent adverse events within Standardized MedDRA Queries/Preferred Terms in patients with and without aura. (DOCX 39 kb) [file 10194_2019_1044_MOESM1_ESM.docx]

# **Additional file 1**

# **SUPPLEMENTAL MATERIALS**

## **Safety and tolerability**

### ***Potential and likely CV adverse events***

Overall, there was a low proportion of lasmiditan- and placebo-treated patients that reported potential CV AEs (Supplemental Table 2). The number and percentage of patients with at least 1 potential CV AE were significantly higher in lasmiditan-treated patients (*n* = 97 [3.1%]) than in placebo-treated patients (*n* = 18 [1.4%]) (*p* = 0.002). This was also the case in the Cardiomyopathy (*p* = 0.01) and CNS vascular disorders (*p* = 0.03) SMQs, but there was no significant difference in the other 7 SMQs or the abdominal pain PTs. Supplemental Table 3 shows the resultant percentages of likely CV AEs after medical case reviews. No statistically significant differences were observed between the lasmiditan- and placebo-treated patients in any of the 9 SMQs or the abdominal pain PTs for likely CV AEs.

When analyzed by the presence of CVRFs, likely CV AEs were not statistically different in those treated with lasmiditan versus placebo in either the presence or absence of any CVRFs. In both treatment groups (lasmiditan vs. placebo), likely CV AEs were most frequent (*n* = 47 [1.5%] vs. *n* = 14 [1.1%]) in patients with ≥ 1 CVRF.

# **Supplemental Tables**

**Supplemental Table 1** Summary of events excluded from likely adverse events and the associated Standardized MedDRA Queries/Preferred Terms

| **Patient case descriptions** | **Reported term/ Preferred Term** | **SMQ or Preferred Term** |
| --- | --- | --- |
|  |  |  |
| Case 1: Syncope 16 hours after 50 mg lasmiditan with no history of or comorbid cardiac event and no concomitant CV medications | Syncope/Syncope | Cardiac arrhythmias  Cardiomyopathy  Torsade de pointes/QT prolongation |
| Case 2: Vasovagal syncope after 50 mg lasmiditan with no history of or comorbid cardiac event and no concomitant CV medications | Vasovagal syncope/Syncope | Cardiac arrhythmias  Cardiomyopathy  Torsade de pointes/QT prolongation |
| Case 3: Edema of the lower leg after 100 mg lasmiditan with no history of or comorbid cardiac event and no concomitant CV medications; had history of ovarian cancer; no treatment was given for edema | Tibilateral lower leg edema/Edema peripheral | Cardiac failure |
| Case 4: Peripheral swelling of right 4th toe after 100 mg lasmiditan with no history of or comorbid cardiac event and no concomitant CV medications | Right foot 4th toe swelling/Peripheral swelling | Cardiac failure |
| Case 5: Dyspnea after 200 mg lasmiditan with no history of or comorbid cardiac event and no concomitant CV medications | Shortness of breath/Dyspnea | Cardiomyopathy  Pulmonary HTN |
| Case 6: Dyspnea after 100 mg lasmiditan with no history of or comorbid cardiac event and no concomitant CV medications | Shortness of breath/Dyspnea | Cardiomyopathy  Pulmonary HTN |
| Case 7: Dyspnea after 100 mg lasmiditan with no history of or comorbid cardiac event and no concomitant CV medications | Shortness of breath/Dyspnea | Cardiomyopathy  Pulmonary HTN |
| Case 8: Dyspnea after 200 mg lasmiditan with no history of or comorbid cardiac event and no concomitant CV medications; had history of panic disorder | Shortness of breath/Dyspnea | Cardiomyopathy  Pulmonary HTN |
| Case 9: Mental status change after 200 mg lasmiditan with no history of or comorbid cardiac event and no concomitant CV medications | Altered mental status/Mental status changes | Cardiomyopathy |
| Case 10: Dysarthria after 100 mg lasmiditan with no history of or comorbid cardiac event and no concomitant CV medications | Slurred speech/Dysarthria | CNS vascular disorders |
| Case 11: Dysarthria after 200 mg lasmiditan with no history of or comorbid cardiac event and no concomitant CV medications | Dysarthria/Dysarthria | CNS vascular disorders |
| Case 12: Dysarthria after 200 mg lasmiditan with no history of or comorbid cardiac event and no concomitant CV medications; had history of GERD | Dysarthria/Dysarthria | CNS vascular disorders |
| Case 13: Dysarthria after 200 mg lasmiditan with no history of or comorbid cardiac event and no concomitant CV medications; had history of GERD | Slurred speech/Dysarthria | CNS vascular disorders |
| Case 14: Dysarthria after 200 mg lasmiditan with no history of or comorbid cardiac event and no concomitant CV medications | Slurred speech/Dysarthria | CNS vascular disorders |
| Case 15: Dysarthria after 100 mg lasmiditan with no history of or comorbid cardiac event and no concomitant CV medications | Slurred speech/Dysarthria | CNS vascular disorders |
| Case 16: Dysarthria after 200 mg lasmiditan with no history of or comorbid cardiac event and no concomitant CV medications | Dysarthria/Dysarthria | CNS vascular disorders |
| Case 17: Dysarthria after 200 mg lasmiditan with no history of or comorbid cardiac event and no concomitant CV medications; had concomitant paresthesia and dizziness | Slurred speech/Dysarthria | CNS vascular disorders |
| Case 18: Dysarthria after 200 mg lasmiditan with no history of or comorbid cardiac event and no concomitant CV medications; had concomitant dizziness, fatigue, paresthesia, tremor, and headache | Dysarthria/Dysarthria | CNS vascular disorders |
| Case 19: Aphasia after 200 mg lasmiditan with no history of or comorbid cardiac event and no concomitant CV medications | Amnesic dysphasia/Aphasia | CNS vascular disorders |
| Case 20: Dysarthria after 100 mg lasmiditan with no history of or comorbid cardiac event and no concomitant CV medications | Slurred speech/Dysarthria | CNS vascular disorders |
| Case 21: Abdominal pain after 100 mg lasmiditan with no history of or comorbid cardiac event and no concomitant CV medications | Abdominal cramping/Abdominal pain | Any abdominal pain |
| Case 22: Abdominal pain after placebo with no history of or comorbid cardiac event and no concomitant CV medications | Upper abdominal cramping/Abdominal pain | Any abdominal pain |
| Case 23: Abdominal pain after placebo with no history of or comorbid cardiac event and no concomitant CV medications | Stomach ache/Abdominal pain upper | Any abdominal pain |
| Case 24: Abdominal pain after 100 mg lasmiditan with no history of or comorbid cardiac event and no concomitant CV medications | Stomach ache/Abdominal pain upper | Any abdominal pain |
| Case 25: Abdominal pain after 100 mg lasmiditan with no history of or comorbid cardiac event and no concomitant CV medications | Abdominal pain/Abdominal pain | Any abdominal pain |
| Case 26: Abdominal pain after 100 mg lasmiditan with no history of or comorbid cardiac event and no concomitant CV medications; had concomitant vomiting; had history of GERD and IBS | Abdominal cramps/Abdominal pain upper | Any abdominal pain |
| Case 27: Abdominal pain after 100 mg lasmiditan with no history of or comorbid cardiac event and no concomitant CV medications; responded to treatment with peptic ulcer medication | Stomach pain/Abdominal pain upper | Any abdominal pain |
| Case 28: Abdominal pain after 100 mg lasmiditan with no history of or comorbid cardiac event and no concomitant CV medications; responded to treatment with antacid medication | Stomach pain/Abdominal pain upper | Any abdominal pain |
| Case 29: Abdominal pain after 50 mg lasmiditan with no history of or comorbid cardiac event and no concomitant CV medications; had history of GERD | Stomach ache/Abdominal pain upper | Any abdominal pain |
| Case 30: Abdominal pain after 50 mg lasmiditan with no history of or comorbid cardiac event and no concomitant CV medications | Abdominal pain/Abdominal pain | Any abdominal pain |
| Case 31: Abdominal pain after 200 mg lasmiditan with no history of or comorbid cardiac event and no concomitant CV medications; had history of constipation and UC | Stomach ache/Abdominal pain upper | Any abdominal pain |
| Case 32: Abdominal pain after 100 mg lasmiditan with no history of or comorbid cardiac event and no concomitant CV medications | Abdominal cramping/Abdominal pain | Any abdominal pain |
| Case 33: Abdominal pain after 50 mg lasmiditan with no history of or comorbid cardiac event and no concomitant CV medications; had concurrent ovarian cyst rupture | Abdominal pain/Abdominal pain | Any abdominal pain |
| Case 34: Abdominal pain after 50 mg lasmiditan with no history of or comorbid cardiac event and no concomitant CV medications; had concurrent UTI | Right lower quadrant abdominal pain/Abdominal pain lower | Any abdominal pain |
| Case 35: Abdominal pain after 200 mg lasmiditan with no history of or comorbid cardiac event and no concomitant CV medications; had history of gastric ulcer and concurrent metrorrhagia | Stomach ache/Abdominal pain upper | Any abdominal pain |
| Case 36: Abdominal pain after 100 mg lasmiditan with no history of or comorbid cardiac event and no concomitant CV medications; had history of multiple GI and gynecological conditions | Abdominal cramps/Abdominal pain | Any abdominal pain |
| Case 37: Abdominal pain after 100 mg lasmiditan with no history of or comorbid cardiac event and no concomitant CV medications | Stomach pain/Abdominal pain upper | Any abdominal pain |
| Case 38: Abdominal pain after 50 mg lasmiditan with no history of or comorbid cardiac event and no concomitant CV medications | Stomach pain/Abdominal pain upper | Any abdominal pain |
| Case 39: Abdominal pain after 50 mg lasmiditan with no history of or comorbid cardiac event and no concomitant CV medications | Stomach pain/Abdominal pain upper | Any abdominal pain |
| Case 40: Abdominal pain after 200 mg lasmiditan with no history of or comorbid cardiac event and no concomitant CV medications | Abdominal pain/Abdominal pain | Any abdominal pain |
| Case 41: Abdominal pain after 50 mg lasmiditan with no history of or comorbid cardiac event and no concomitant CV medications; had history of asthma and concurrent anxiety | Stomach pain/Abdominal pain upper | Any abdominal pain |
| Case 42: Abdominal pain after 100 mg lasmiditan with no history of or comorbid cardiac event and no concomitant CV medications | Abdominal pain/Abdominal pain | Any abdominal pain |
| Case 43: Abdominal pain after 200 mg lasmiditan with no history of or comorbid cardiac event and no concomitant CV medications | Stomach ache/Abdominal pain upper | Any abdominal pain |
| Case 44: Abdominal pain after 200 mg lasmiditan with no history of or comorbid cardiac event and no concomitant CV medications; had concurrent diarrhea | Stomach cramping/Abdominal pain upper | Any abdominal pain |

*AE* adverse event, *CNS* central nervous system, *CV*cardiovascular, *GERD* gastroesophageal reflux disease, *GI*gastrointestinal, *IBS* irritable bowel syndrome, *LTN* lasmiditan, *MedDRA*Medical Dictionary for Drug Regulatory Activities, *PT*Preferred Term, *SMQ* Standardized MedDRA Query, *UC* ulcerative colitis, *UTI* urinary tract infection.

Note: Potential CV AEs are based on broad and narrow terms in the SMQs Cardiac arrhythmias, Cardiac failure, Cardiomyopathy, CNS vascular disorders, Embolic and thrombotic events, Hypertension, Ischemic heart disease, Pulmonary hypertension, and Torsade de pointes/QT prolongation and the PTs abdominal pain, abdominal pain upper, and abdominal pain lower.

Note: Any abdominal pain (PT) consists of the PTs abdominal pain, abdominal pain upper, and abdominal pain lower.

MedDRA version 21.0.

**Supplemental Table 2** Summary and analysis of potential cardiovascular adverse events within Standardized MedDRA Queries/Preferred Terms

| **Standardized MedDRA Query**  **Preferred Term** | **Placebo**  **(*N* = 1,262)** | **All LTN**  **(*N* = 3,177)** | **Comparison between all LTN vs. placebo** | | |
| --- | --- | --- | --- | --- | --- |
|  | ***n* (%) [adj %]** | ***n* (%) [adj %]** | **OR^a^** | **95% CI^a^** | ***p* value^b^** |
| Patients with at least 1 potential CV AE | 18 (1.4) [1.4] | 97 (3.1) [3.1] | 2.21 | (1.33, 3.68) | **0.002** |
| Cardiac arrhythmias (SMQ) | 8 (0.6) [0.6] | 40 (1.3) [1.3] | 1.98 | (0.92, 4.25) | 0.08 |
| Cardiac failure (SMQ) | 1 (0.1) [0.1] | 3 (0.1) [0.1] | 1.25 |  | 0.86 |
| Cardiomyopathy (SMQ) | 2 (0.2) [0.2] | 25 (0.8) [0.8] | 5.08 | (1.20, 21.50) | **0.01** |
| CNS vascular disorders (SMQ) | 0 (0.0) [0.0] | 11 (0.3) [0.3] |  |  | **0.03** |
| Embolic and thrombotic events (SMQ) | 0 (0.0) [0.0] | 1 (0.0) [0.0] |  |  | 0.56 |
| Hypertension (SMQ) | 3 (0.2) [0.3] | 13 (0.4) [0.4] | 1.67 | (0.48, 5.73) | 0.40^c^ |
| Ischemic heart disease (SMQ) | 0 (0.0) [0.0] | 2 (0.1) [0.1] |  |  | 0.32 |
| Pulmonary hypertension (SMQ) | 1 (0.1) [0.1] | 5 (0.2) [0.2] | 2.37 | (0.27, 20.62) | 0.42 |
| Torsade de pointes/QT prolongation (SMQ) | 0 (0.0) [0.0] | 6 (0.2) [0.2] |  |  | 0.14 |
| Any abdominal pain (PT) | 4 (0.3) [0.3] | 22 (0.7) [0.7] | 2.28 | (0.76, 6.81) | 0.14^c^ |

^a^Mantel-Haenszel OR stratified by study and 95% CI (CI calculated if ≥ 4 events in numerator and ≥ 1 event in denominator).

^b^*p* values are from Cochran-Mantel-Haenszel test of general association stratified by study. Bold indicates a *p* value < 0.05.

^c^*p* value < 0.1 for heterogeneity test of ORs across studies (assessed using the Breslow Day test).

*adj %* study size adjusted percentage, *AE* adverse event, *CI* confidence interval, *CNS* central nervous system, *CV* cardiovascular, *LTN* lasmiditan, *MedDRA* Medical Dictionary for Drug Regulatory Activities, *N* number of patients in the analysis population, *n* number of patients within each specific category; *OR* odds ratio, *PT* Preferred Term, *SMQ* Standardized MedDRA Query.

Note: Potential CV AEs are based on broad and narrow terms in the SMQs Cardiac arrhythmias, Cardiac failure, Cardiomyopathy, CNS vascular disorders, Embolic and thrombotic events, Hypertension, Ischemic heart disease, Pulmonary hypertension, and Torsade de pointes/QT prolongation and the PTs abdominal pain, abdominal pain upper, and abdominal pain lower.

Note: Any abdominal pain (PT) consists of the PTs abdominal pain, abdominal pain upper, and abdominal pain lower.

MedDRA version 21.0.

**Supplemental Table 3** Summary and analysis of likely cardiovascular adverse events within Standardized MedDRA Queries/Preferred Terms

| **Standardized MedDRA Query**  **Preferred Term** | **Placebo**  **(*N* = 1,262)** | **All LTN**  **(*N* = 3,177)** | **Comparison between all LTN vs. placebo** | | |
| --- | --- | --- | --- | --- | --- |
|  | ***n* (%) [adj %]** | ***n* (%) [adj %]** | **OR^a^** | **95% CI^a^** | ***p* value^b^** |
| Patients with at least 1 likely CV AE | 16 (1.3) [1.2] | 55 (1.7) [1.7] | 1.40 | (0.79, 2.45) | 0.25 |
| Cardiac arrhythmias (SMQ) | 8 (0.6) [0.6] | 38 (1.2) [1.2] | 1.89 | (0.88, 4.07) | 0.10 |
| Cardiac failure (SMQ) | 1 (0.1) [0.1] | 1 (0.0) [0.0] | 0.50 |  | 0.62 |
| Cardiomyopathy (SMQ) | 2 (0.2) [0.2] | 18 (0.6) [0.6] | 3.65 | (0.84, 15.79) | 0.06 |
| Embolic and thrombotic events (SMQ) | 0 (0.0) [0.0] | 1 (0.0) [0.0] |  |  | 0.56 |
| Hypertension (SMQ) | 3 (0.2) [0.3] | 13 (0.4) [0.4] | 1.67 | (0.48, 5.73) | 0.40^c^ |
| Ischemic heart disease (SMQ) | 0 (0.0) [0.0] | 2 (0.1) [0.1] |  |  | 0.32 |
| Pulmonary hypertension (SMQ) | 1 (0.1) [0.1] | 1 (0.0) [0.0] | 0.50 |  | 0.62 |
| Torsade de pointes/QT prolongation (SMQ) | 0 (0.0) [0.0] | 4 (0.1) [0.1] |  |  | 0.22 |
| Any abdominal pain (PT) | 2 (0.2) [0.1] | 0 (0.0) [0.0] | 0.00 |  | 0.05 |

^a^Mantel-Haenszel OR stratified by study and 95% CI (CI calculated if ≥ 4 events in numerator and ≥ 1 event in denominator).

^b^*p* values are from Cochran-Mantel-Haenszel test of general association stratified by study.

^c^*p* value < 0.1 for heterogeneity test of ORs across studies (assessed using the Breslow Day test).

*adj %* study size adjusted percentage, *AE* adverse event, *CI*confidence interval, *CNS* central nervous system, *CV* cardiovascular, *LTN*lasmiditan, *MedDRA* Medical Dictionary for Drug Regulatory Activities, *N* number of patients in the analysis population, *n* number of patients within each specific category, *OR* odds ratio, *PT* Preferred Term, *SMQ* Standardized MedDRA Query.

Note: Likely CV AEs are from medical review out of potential CV AEs that are selected based on broad and narrow terms in the SMQs Cardiac arrhythmias, Cardiac failure, Cardiomyopathy, CNS vascular disorders, Embolic and thrombotic events, Hypertension, Ischemic heart disease, Pulmonary hypertension, and Torsade de pointes/QT prolongation and the PTs abdominal pain, abdominal pain upper, and abdominal pain lower.

Note: Any abdominal pain (PT) consists of the PTs abdominal pain, abdominal pain upper, and abdominal pain lower.

MedDRA version 21.0.

**Supplemental Table 4** Summary of likely cardiovascular adverse events within Standardized MedDRA Queries/Preferred Terms in patients with and without aura

| **Standardized MedDRA Query**  **Preferred Term** | **Placebo** | | **All LTN** | |
| --- | --- | --- | --- | --- |
|  | **No Aura (*N* = 737)** | **Aura (*N* = 515)** | **No Aura (*N* = 1,911)** | **Aura (*N* = 1,244)** |
|  | ***n* (%)** | ***n* (%)** | ***n* (%)** | ***n* (%)** |
| Patients with at least 1 likely CV AE | 4 (0.5) | 10 (1.9) | 30 (1.6) | 24 (1.9) |
| Cardiac arrhythmias (SMQ) | 2 (0.3) | 5 (1.0) | 20 (1.0) | 17 (1.4) |
| Cardiac failure (SMQ) | 0 (0.0) | 1 (0.2) | 0 (0.0) | 1 (0.1) |
| Cardiomyopathy (SMQ) | 1 (0.1) | 1 (0.2) | 12 (0.6) | 5 (0.4) |
| Embolic and thrombotic events (SMQ) | 0 (0.0) | 0 (0.0) | 0 (0.0) | 1 (0.1) |
| Hypertension (SMQ) | 1 (0.1) | 2 (0.4) | 9 (0.5) | 4 (0.3) |
| Ischemic heart disease (SMQ) | 0 (0.0) | 0 (0.0) | 1 (0.1) | 1 (0.1) |
| Pulmonary hypertension (SMQ) | 0 (0.0) | 0 (0.0) | 1 (0.1) | 0 (0.0) |
| Torsade de pointes/QT prolongation (SMQ) | 0 (0.0) | 0 (0.0) | 1 (0.1) | 3 (0.2) |
| Any abdominal pain (PT) | 0 (0.0) | 2 (0.4) | 0 (0.0) | 0 (0.0) |

*AE* adverse event, *CNS* central nervous system, *CV*cardiovascular, *LTN*lasmiditan, *MedDRA* Medical Dictionary for Drug Regulatory Activities, *N* number of patients in the analysis population, *n* number of patients within each specific category, *PT* Preferred Term, *SMQ* Standardized MedDRA Query.

Note: Likely CV AEs are from medical review out of potential CV AEs that are selected based on broad and narrow terms in the SMQs Cardiac arrhythmias, Cardiac failure, Cardiomyopathy, CNS vascular disorders, Embolic and thrombotic events, Hypertension, Ischemic heart disease, Pulmonary hypertension, and Torsade de pointes/QT prolongation and the PTs abdominal pain, abdominal pain upper, and abdominal pain lower.

Note: Any abdominal pain (PT) consists of the PTs abdominal pain, abdominal pain upper, and abdominal pain lower.

Note: Patients were categorized based on International Headache Society Diagnostic Criteria: Migraine with Aura (Yes/No).

MedDRA version 21.0.

**Supplemental Table 5** Summary of likely cardiovascular treatment-emergent adverse events within Standardized MedDRA Queries/Preferred Terms in patients with and without aura

| **Standardized MedDRA Query**  **Preferred Term** | **Placebo** | | **All LTN** | |
| --- | --- | --- | --- | --- |
|  | **No Aura (*N* = 737)** | **Aura (*N* = 515)** | **No Aura (*N* = 1,911)** | **Aura (*N* = 1,244)** |
|  | ***n* (%)** | ***n* (%)** | ***n* (%)** | ***n* (%)** |
| Patients with at least 1 likely CV TEAE | 1 (0.1) | 3 (0.6) | 18 (0.9) | 11 (0.9) |
| Cardiac arrhythmias (SMQ) | 1 (0.1) | 2 (0.4) | 16 (0.8) | 10 (0.8) |
| Cardiomyopathy (SMQ) | 0 (0.0) | 1 (0.2) | 9 (0.5) | 4 (0.3) |
| Hypertension (SMQ) | 0 (0.0) | 0 (0.0) | 2 (0.1) | 1 (0.1) |
| Torsade de pointes/QT prolongation (SMQ) | 0 (0.0) | 0 (0.0) | 1 (0.1) | 0 (0.0) |
| Any abdominal pain (PT) | 0 (0.0) | 1 (0.2) | 0 (0.0) | 0 (0.0) |

*AE* adverse event, *CNS* central nervous system, *CV* cardiovascular, *LTN* lasmiditan, *MedDRA* Medical Dictionary for Drug Regulatory Activities, *N* number of patients in the analysis population, *n* number of patients within each specific category, *PT* Preferred Term, *SMQ* Standardized MedDRA Query, *TEAE* treatment-emergent adverse event

Note: Likely CV AEs are from medical review out of potential CV AEs that are selected based on broad and narrow terms in the SMQs Cardiac arrhythmias, Cardiac failure, Cardiomyopathy, CNS vascular disorders, Embolic and thrombotic events, Hypertension, Ischemic heart disease, Pulmonary hypertension, and Torsade de pointes/QT prolongation and the PTs abdominal pain, abdominal pain upper, and abdominal pain lower.

Note: Any abdominal pain (PT) consists of the PTs abdominal pain, abdominal pain upper, and abdominal pain lower.

Note: Patients were categorized based on International Headache Society Diagnostic Criteria: Migraine with Aura (Yes/No).

MedDRA version 21.0.
